# Knowledge, attitudes, perceptions, and practices toward antiretroviral therapy among people living with HIV in secondary healthcare facilities in Lagos State, Nigeria: a multicenter cross-sectional study

**DOI:** 10.1186/s12889-025-25629-1

**Published:** 2025-12-30

**Authors:** Qudus A. Ojomo, Ukamaka G. Okafor, Titilayo A. Onedo, Margaret O. Adedapo, Dorcas Nyalas-Omeire, Rahmotallah M. Babalola, Afusat Adesina, Patricia T. Odunuga, Jonathan Ikokwu, Abigail I. Okonu

**Affiliations:** 1 Pharmacy Department, Lagos State University Teaching Hospital, Ikeja, Lagos Nigeria; 2 Department of Global Health and Bioethics, EUCLID University, Bangui, Central African Republic; 3 Pharmacy Department, National Orthopaedic Hospital, Igbobi, Lagos Nigeria; 4 Logistics Management Unit, Lagos State Ministry of Health, Alausa, Ikeja, Lagos Nigeria; 5 Pharmacy Unit, University of Lagos Medical Centre, Akoka, Lagos Nigeria; 6 Private-Public Partnerships Unit, John Snow Incorporation, Abuja, Nigeria; 7 Inspection, Monitoring and Quality Assurance Unit, Pharmacy Council of Nigeria, Lagos Zonal Office, Lagos, Nigeria; 8 Expert Pool Unit, Africa Resource Center for Excellence in Supply Chain Management, Lagos, Nigeria; 9 Association of Hospital and Administrative Pharmacists of Nigeria (AHAPN), Lagos, Nigeria

**Keywords:** HIV, PLHIV, ART, Knowledge, Attitude, Perception, Practice

## Abstract

**Background:**

Despite global progress against HIV/AIDS through widespread antiretroviral therapy (ART) access, treatment success still hinges on adherence, shaped by the knowledge, attitudes, perceptions, and practices of people living with HIV (PLHIV). In densely populated Lagos, Nigeria, where the HIV burden remains high, this study assessed factors influencing ART adherence to inform future strategies for improving treatment outcomes.

**Methodology:**

A multicenter, descriptive, cross-sectional study design was employed. Data were collected using a structured, self-administered questionnaire administered to consenting individuals living with HIV who had been receiving antiretroviral therapy for at least three months.

600 pre-tested questionnaires were administered. Of these, 513 were completed and analyzed, resulting in a response rate of 85.5%. Respondents are PLHIV attending antiretroviral clinics across six selected secondary healthcare facilities in Lagos. Data collection took place between February 2024 and April 2024. Descriptive statistics were generated using SPSS version 23. Pearson correlation and multiple linear regression were conducted to examine relationships among variables.

**Results:**

The mean age of respondents was 43 years, with 74.9% (384/513) being female. Approximately 94% of participants demonstrated satisfactory knowledge of ART. Additionally, 81.1% exhibited satisfactory attitudes toward treatment, while 68.4% had poor perceptions of their medications. Nearly half of the respondents (45.4%, 233/513) reported storing their medicines in a concealed location within their residence.

A significant but weak negative correlation was found between knowledge and perception of PLHIV towards ART (r(513) = -0.129, *p* = 0.003). No significant correlation was found between their knowledge and attitude towards ART (r(513) = 0.018, *p* = 0.683).

Multiple linear regression analysis revealed a statistically significant negative association between respondents' perception of ART and their knowledge of ART (β = –0.609, *p* = 0.003), indicating that less favorable perceptions were associated with lower levels of knowledge about ART.

**Conclusion:**

People living with HIV in Lagos State demonstrate satisfactory knowledge and attitudes toward antiretroviral therapy; however, negative perceptions about their medications remain prevalent. HIV-related stigma continues to influence key behavioral practices, such as medication concealment and nondisclosure of HIV status. Strengthening patient-centered education and stigma-reduction efforts may enhance ART adherence and improve long-term treatment outcomes in this population.

**Supplementary Information:**

The online version contains supplementary material available at 10.1186/s12889-025-25629-1.

## Introduction

The Human Immunodeficiency Virus (HIV) has been a source of global public health challenges, accounting for 40.1 million deaths since its epidemic [1]. The progression from being infected with the virus to a full-blown Acquired Immune Deficiency Syndrome (AIDS) has been a global crisis, negatively impacting the health of many [2]. About 38.4 million people were living with the disease globally as of 2021, of which 25.6 million were in Africa [3].

HIV/AIDS remains a significant global health challenge, particularly in sub-Saharan Africa, which bears the highest burden of the disease. Nigeria, the most populous country in Africa, accounts for a substantial proportion of the region’s HIV cases [4]. Despite ongoing efforts to mitigate the impact of the epidemic, the country continues to face challenges related to the prevention, diagnosis, and management of HIV [5].

Antiretroviral therapy (ART) is the standard treatment for HIV and involves a combination of drugs that suppress viral replication and improve immune function. The World Health Organization recommends initiating ART in all people living with HIV, regardless of clinical stage or CD4 count [6]. ART drugs are classified based on the stage of the HIV life cycle they target.

Antiretroviral therapy has been pivotal in transforming HIV from a fatal disease to a manageable chronic condition [7], thereby improving the quality of life and life expectancy of people living with HIV (PLHIV). However, the success of ART programs depends significantly on the knowledge, attitudes, perceptions, and practices (KAPP) of PLHIV toward treatment [8, 9].

In Nigeria, the integration of ART into the public health system has been supported by interventions aimed at improving access, adherence, and retention in care [10, 11]. Secondary healthcare facilities, which often serve as the first point of contact for PLHIV, play a critical role in delivering comprehensive HIV services [12]. While ART has transformed HIV into a manageable chronic condition, its effectiveness depends not only on healthcare infrastructure but also on the knowledge, attitudes, perceptions, and practices of PLHIV regarding treatment [12].

Knowledge about ART is crucial for adherence and achieving positive health outcomes [13]. It encompasses understanding the purpose of the therapy, the importance of consistent usage, potential side effects, and the long-term benefits of viral suppression. Without adequate knowledge, PLHIV may struggle to adhere to treatment, increasing the risk of drug resistance and compromising public health efforts to control the epidemic [13]. Additionally, attitudes and perceptions, such as stigma, fear of side effects, or mistrust of healthcare systems, can significantly influence treatment adherence. Negative attitudes towards ART or misconceptions about its efficacy may lead to poor health-seeking behaviors or treatment interruptions [14].

Lagos State, recognized as Nigeria’s principal economic and demographic center, provides a distinctive environment for investigating the KAPP of PLHIV to antiretroviral therapy [15]. With its heterogeneous population and a combination of urban and peri-urban settings, the state’s healthcare system is subject to significant demands, particularly in addressing the needs of PLHIV [16]. Secondary healthcare facilities constitute a crucial component of HIV service delivery in Lagos, offering ART alongside counseling and psychosocial support. Although previous studies have explored aspects of knowledge, attitudes, and practices related to antiretroviral therapy among PLHIV in Nigeria [17, 18], there remains limited evidence specifically focusing on the comprehensive interplay of knowledge, attitudes, perceptions, and practices in secondary healthcare settings in Lagos State. This study seeks to fill that gap by providing context-specific insights into these domains and their associations.

To address this knowledge gap, the present study investigates the knowledge, attitudes, perceptions, and practices of people living with HIV in Lagos State, southwestern Nigeria, in relation to their use of antiretroviral therapy.

The findings of this study will contribute to the growing body of evidence on HIV care and treatment in Nigeria, providing insights for policymakers, healthcare providers, and community organizations.

## Methods

### Research design

This study was a multicenter, descriptive, and cross-sectional questionnaire-based survey of PLHIV’s knowledge, attitudes, perceptions, and practices towards antiretroviral therapy among clients of secondary healthcare facilities in Lagos State, Nigeria. It was conducted between February 2024 and April 2024.

### Sampling procedure

**Fig. 1:**
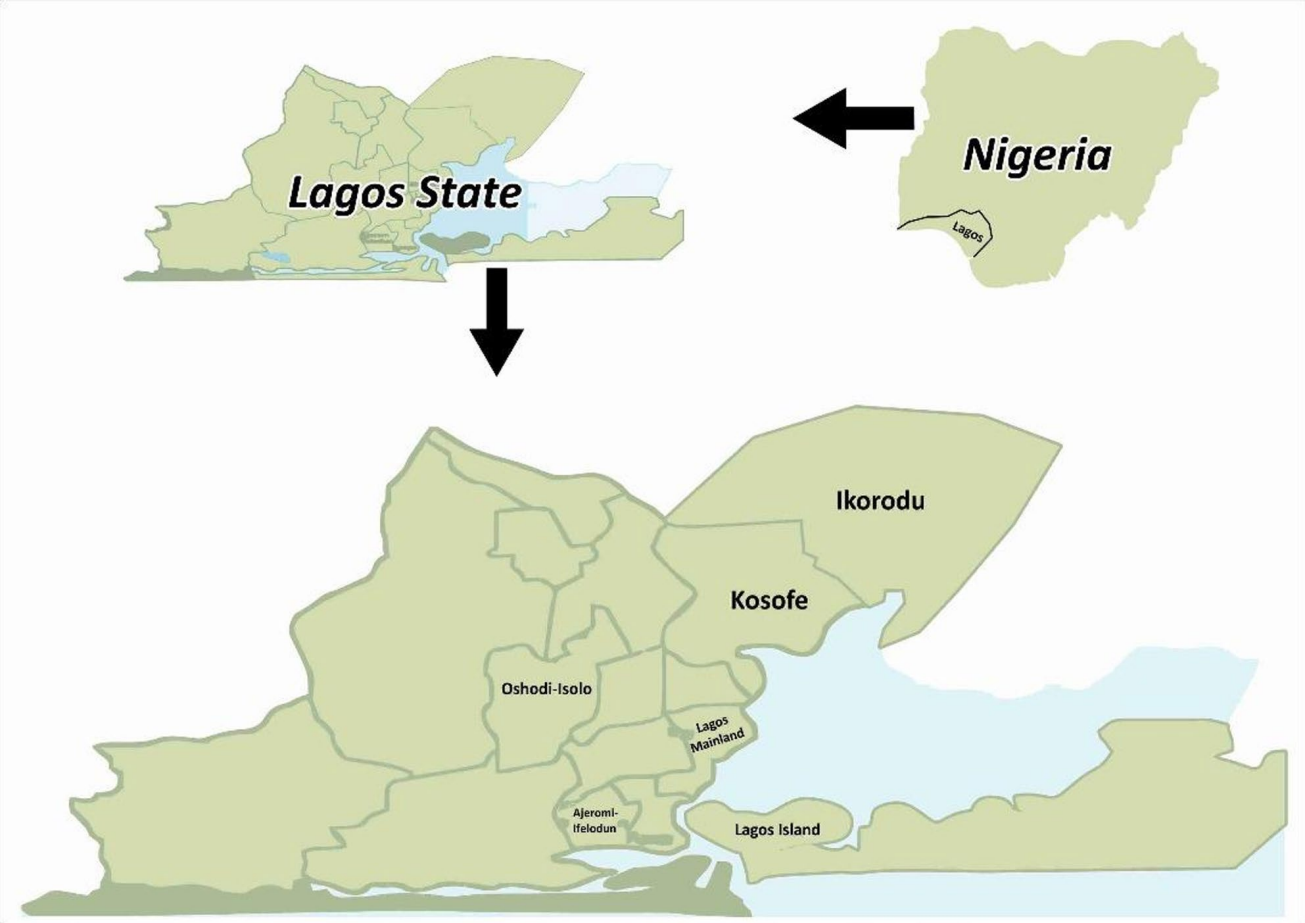
Lagos State map highlighting the study sites local government areas (LGAs): Kosofe, Lagos Mainland, Lagos Island, Oshodi-Isolo, Ikorodu, and Ajeromi-Ifelodun

The study was conducted in six hospitals located across Lagos State, Nigeria. These hospitals were purposively selected to ensure representation across diverse local government areas (LGAs) within the state (Fig. 1). The selected hospitals include:

1. General Hospital, Lagos (Lagos Island LGA).
2. General Hospital, Ikorodu (Ikorodu LGA).
3. Ajeromi General Hospital (Ajeromi-Ifelodun LGA).
4. Isolo General Hospital (Oshodi/Isolo LGA).
5. Gbagada General Hospital (Kosofe LGA).
6. Mainland Hospital, Yaba (Lagos Mainland LGA).

To recruit participants, a multi-stage sampling technique was employed. In the first stage, the selected hospitals were stratified according to their respective LGAs. In the second stage, within each hospital, departments relevant to the study objectives—particularly outpatient clinics such as antiretroviral therapy (ART) clinics, pharmacy departments, and other units—were purposively identified for participant selection. This approach ensured the inclusion of individuals receiving or interacting with ART services, aligning with the study’s focus. In the final stage, consecutive sampling was used to recruit eligible participants. All PLHIV who presented at the ART clinic during the study period and met the inclusion criteria were invited to participate. Those who provided informed consent and had been on antiretroviral therapy for a minimum of three months were enrolled. This approach was adopted due to practical considerations related to patient flow and clinic operations. Sample size determination was guided by statistical considerations to ensure adequate power for detecting meaningful differences or associations.

### Sample size determination

It was estimated that about 18,000 PLHIV would attend the clinics of the selected secondary healthcare institutions within the study duration (three months). Thus, the minimum sample size was calculated as 391, as shown below using the Taro Yamane sample size formula [19].

$$\begin{aligned} & \:n=\frac{N}{1+N{e}^{2}}\:n=\frac{N}{1+N{e}^{2}}=\:n=\frac{\text{18,000}}{1+\text{18,000}*{0.05}^{2}}\:\\& n=\frac{\text{18,000}}{1+\text{18,000}*{0.05}^{2}} \end{aligned}$$

*n* ≈ 391.

n = minimum sample size.

N = population size = 18,000 (for 3 months).

𝑒 = error (0.05), reliability level 95%.

### Study sites

The study was conducted in six secondary healthcare facilities across different Local Government Areas (LGAs) in Lagos State, Nigeria. These hospitals were purposively selected based on their geographic distribution, patient volume, and their provision of comprehensive ART services. All sites are public, government-owned secondary hospitals offering outpatient and inpatient services.

- General Hospital, Lagos (Lagos Island LGA) is a 250-bed facility with an average monthly ART clinic attendance of approximately 800 patients.
- Ajeromi General Hospital (Ajeromi-Ifelodun LGA) has 133 beds, and its ART clinic serves around 700 patients monthly.
- General Hospital, Ikorodu (Ikorodu LGA) is a 285-bed hospital, with an average monthly ART attendance of 1,016 patients.
- Mainland Hospital, Yaba (Lagos Mainland LGA), a hospital specializing in infectious diseases, is an 82-bed facility that serves an average of 500 ART patients monthly.
- General Hospital, Isolo (Oshodi-Isolo LGA) is a 175-bed hospital, with approximately 995 patients attending the ART clinic each month.
- Gbagada General Hospital (Kosofe LGA) is a 192-bed facility seeing an average of 350 patients monthly in its ART clinic.

All selected hospitals participate in Nigeria’s national HIV program and maintain routine records through the National Health Management Information System (NHMIS). The variation in facility size and patient volume provided a diverse and representative sample of PLHIV accessing ART services in Lagos State.

### Study tool

A structured and self-administered paper questionnaire was developed for this study, adapted from a previously validated Knowledge, Attitude, and Practice (KAP) instrument used in a similar population [20], and modified to suit the local context (see Supplementary Data 1). The draft tool was piloted among 25 PLHIV who were excluded from the final participants to establish face validity and detect any ambiguities. Minor revisions were made based on participant feedback and expert review. The final instrument demonstrated acceptable internal consistency with a Cronbach’s alpha of 0.67.

The questionnaire consisted of four sections:

1. Socio-demographic characteristics—including gender, marital status, age, residence, sexual orientation, occupation, education level, living arrangements, ART duration, and HIV status disclosure.
2. Knowledge of ART assessed participants’ awareness of their medication regimen, dosage frequency, treatment duration, intended effects on viral load and CD4 count, and purpose of ART.
3. Attitude and perception of ART – explored beliefs about ART effectiveness, perceived harm or burden, alternative treatments, and emotional barriers such as shame or therapy fatigue.
4. Practice concerning ART – assessed storage methods, medication adherence, missed doses, reminder strategies, and steps taken after losing or discarding medications.

In this study, the constructs of attitude and perception were assessed using a combined set of questions grouped under a single section of the questionnaire. This approach was adopted to minimize participant burden and enhance the usability of the instrument. Nevertheless, conceptual distinctions between attitude and perception were preserved during data analysis. Although the items were presented together, responses were analyzed separately according to their respective conceptual domains (see Supplementary Data 2).

### Scoring system

To quantify responses and classify levels of knowledge, attitudes, and perceptions toward ART, a scoring system was applied. This is a method consistent with prior studies using KAP frameworks [21, 22]. Correct, positive, or favorable responses were assigned a score of 1, and incorrect, negative, or “don’t know” responses were scored 0.

- The knowledge domain included 7 items, with a total possible score of 7. The mean knowledge score was 5.58. Scores ≥ 5.58 were categorized as good knowledge, while scores < 5.58 indicated poor knowledge.
- The attitude domain consisted of 4 items (maximum score = 4), with a mean score of 2.89. Participants scoring ≥ 2.89 were classified as having a positive attitude, and those scoring below this threshold as having a negative attitude.
- The perception domain included 2 items, with a maximum score of 2. The mean perception score was 0.18. Scores ≥ 0.18 were considered good perception, and those < 0.18 were regarded as poor perception.

No composite scoring was applied to the practice domain, which was analyzed descriptively using frequencies and percentages.

### Data collection procedure

Data collection was conducted over three months across six purposively selected secondary healthcare facilities in Lagos State mentioned earlier.

At each facility, data were collected from the outpatient ART clinics, which are the primary service points for people living with HIV. Eligible participants were approached in the clinic waiting areas, and after providing informed consent, were given the questionnaire to complete privately. Research assistants were present on-site to explain the study purpose, provide guidance when needed, and ensure that participants completed the forms independently.

A total of 12 trained research assistants—two per site—coordinated data collection activities. All assistants received standardized training covering the study protocol, informed consent procedures, and ethical considerations, with a focus on maintaining consistency across sites. A data collection guide was used to ensure uniform instructions were given to participants. Supervisors conducted regular reviews of completed questionnaires to ensure completeness and adherence to the protocol. The self-administered format allowed participants to complete the questionnaire at their own pace, typically within 15–20 min.

### Study population

The study population comprised HIV-positive adults (18 years and older) attending the selected health facilities during the study period.

### Inclusion and exclusion criteria

Consecutive sampling was employed in this study. Eligible participants were approached and recruited as they presented to the ART clinics, provided they met the inclusion criteria and gave informed consent. Participants were eligible for inclusion if they were seropositive for HIV, had been receiving ART for at least three months, were 18 years or older, willing to participate, and available during the data collection period. Individuals were excluded if they were critically ill, cognitively impaired (e.g., delirious or mentally incapacitated), or unable to provide informed consent at the time of data collection.

### Ethical approval

This study was conducted following the ethical principles of the Declaration of Helsinki. The ethical clearance and approval letter of this study were obtained from the Lagos State University Teaching Hospital, Health Research, and Ethics Committee with Reference Number LREC/06/10/2191. The participants (PLHIV) were informed of the study’s objectives and potential benefits for the public health system in Nigeria. There were minimal risks to participants as no invasive procedure was involved. Written informed consent of each respondent was sought and obtained before the survey to ensure they were aware of their right to participate or not. Confidentiality and privacy were ensured by omitting participants’ names and identification numbers from the questionnaire.

### Statistical analysis

Data obtained from the study were entered into Microsoft Excel and then analyzed with the Statistical Package for the Social Sciences (SPSS, Version 23). Descriptive analyses were performed using frequencies and percentages and presented in tables and charts where appropriate. Logistic regression, Pearson correlation, and multiple linear regression analysis were used to determine the presence and nature of the relationship between the measured variables. Statistical significance was assumed when *p* ≤ 0.05.

## Result

### Socio-demographic profile of respondents

Out of the 600 copies of the paper questionnaires distributed to participants, 513 were completed and returned, yielding a response rate of 85.5%. After data cleaning to remove incomplete or inconsistent responses, all 513 questionnaires were valid and retained for analysis.

Table 1 shows the socio-demographic characteristics of the respondents. Responses were obtained from six study sites, with the highest number recorded at Isolo General Hospital (19.5%, 100/513). Participants were aged between 18 and 84 years, with a mean age of 43.81 years. Most respondents (32.4%, 166/513) were within 41–50 years of age. More than two-thirds (74.9%, 384/513) of the respondents were female, and 59.6% (306/513) were married. Most respondents (96.3%, 494/513) identified as heterosexual, while 2.3% (12/513) identified as homosexual. A significant proportion (68.8%, 353/513) were employed, while 20.9% (107/513) were unemployed. Over half (56.3%, 289/513) had completed secondary school education, whereas 3.1% (16/513) had no formal education.

**Table 1:** Socio-demographic profile of respondents

Variables	Frequency (N=513)	Percentage (%)
Study Sites		
Mainland Hospital, Yaba	91	17.7
Gbagada General Hospital	82	16.0
Isolo General Hospital	100	19.5
Ajeromi General Hospital	66	12.9
Ikorodu General Hospital	92	17.9
General Hospital Lagos	82	16.0
Age (years):		
18–30 years	78	15.2
31–40 years	125	24.4
41–50 years	166	32.4
51–60 years	101	19.7
61 and above	43	8.4
mean= 43.81, SD= ± 12.319, range= 18 - 84		
Gender		
Male	129	25.1
Female	384	74.9
Marital status		
Single	101	19.7
Married	306	59.6
Divorced	18	3.5
Widowed	63	12.3
Separated	25	4.9
Sexual orientation		
Heterosexual	494	96.3
Homosexual	12	2.3
Bisexual	7	1.4
Profession		
Student	24	4.7
Unemployed	107	20.9
Employed	353	68.8
Retired	28	5.5
Not disclosed	1	0.2
Level of education		
No formal education	16	3.1
Primary	77	15.0
Secondary	289	56.3
Graduate	104	20.3
Postgraduate	26	5.1
Not disclosed	1	0.2
How long have you been using HIV drugs?		
1–2 years	146	28.5
3–5 years	99	19.3
6–10 years	132	25.7
11–20 years	124	24.2
21 years and above	12	2.3
Mean= 7.09 years, SD= ±5.82		
Are you living alone?		
Yes	108	21.1
No	405	78.9
How many people are you living with?		
None	108	21.1
1–3	232	45.2
4–6	163	31.8
7–9	9	1.8
10 and above	1	0.2
Have you disclosed your HIV status to anyone than your healthcare providers?		
Yes	391	76.2
No	122	23.8
Who did you disclose your HIV status to?		
None	122	23.8
Spouse	194	37.8
Mother	62	12.1
Father	12	2.3
Brother	41	8.0
Sister	22	4.3
Friends	4	0.8
Sexual partner	7	1.4
Children	48	9.4
Religious leader	1	0.2
Which area of Lagos do you reside in?		
Urban	175	34.1
Semi-urban	289	56.3
Rural	49	9.6

Regarding duration on HIV medication, 25.7% (132/513) had been taking antiretroviral drugs for 6–10 years, and a small proportion (2.3%, 12/513) had been on treatment for 21 years or more.

Most participants (78.9%, 405/513) reported living with other people, with 45.2% (232/513) living in households of 1–3 individuals. Furthermore, 76.2% (391/513) disclosed their HIV status to individuals other than their healthcare providers, most commonly to their spouses (37.8% 194/513).N

Although the study sites were located across six local government areas with varying socioeconomic contexts, most respondents (56.3%, 289/513) resided in semi-urban areas of Lagos.

### Knowledge of PLHIV on antiretroviral therapy

Table 2 presents the responses of people living with HIV in Lagos State regarding their knowledge of antiretroviral therapy. Approximately one-third of the respondents (33.9%, 174/513) were unable to identify the name of their medication. Among those who did, the majority (53.8%, 276/513) identified it as Tenofovir/Dolutegravir/Lamivudine.

**Table 2:** Knowledge of PLHIV on antiretroviral therapy

Variables	Frequency (N=513)	Percentage (%)
What are the names of your HIV drugs?		
Tenofovir/Dolutegravir/Lamivudine	276	53.8
Abacavir/Lamivudine/Dolutegravir	16	3.1
Tenofovir/Lamivudine/Atazanavir	20	3.9
Abacavir/Lamivudine/Atazanavir	8	1.6
Zidovudine/Lamivudine/Atazanavir	19	3.7
I don’t know it	174	33.9
How many times should you take your HIV drugs in a day?		
Once a day	492	95.9
Twice daily	18	3.5
I don’t know	3	0.6
How long should you take your HIV drugs?		
As long as I live	420	81.9
For some time, only	42	8.2
I don’t know	51	9.9
What is the purpose of HIV drugs?		
To suppress the activity of HIV but not cure	427	83.2
It cures HIV/AIDS	7	1.4
I don’t know	79	15.4
Effect of HIV drugs on viral load		
Increases viral load	70	13.6
Decreases viral load	391	76.2
No effect on viral load	5	1.0
I don’t know	47	9.2
Effect of HIV drugs on CD4+ count		
Increases CD4+ count	234	45.6
Decreases CD4+ count	124	24.2
No effect on CD4+ count	9	1.8
I don’t know	146	28.5
HIV drugs are relatively safe to use		
No	39	7.6
Yes	474	92.4

Most respondents (95.9%, 492/513) reported taking ART once daily, and 81.9% (420/513) correctly indicated that ART should be taken for life. In addition, 83.2% understood that the purpose of ART is to suppress the activity of HIV, not to cure it. A substantial proportion (76.2%, 391/513) were aware that ART reduces viral load, while 9.2% stated that they did not know its effect on viral load.

However, knowledge regarding the effect of ART on CD4 + count was less robust. Fewer than half (45.6%, 234/513) correctly indicated that ART increases CD4 + count, while 24.2% (124/513) gave an incorrect response, and 28.5% (146/513) reported not knowing the effect. A vast majority (92.4%, 474/513) believed that ART is generally safe to use.

Based on the knowledge scoring system described in the methodology, 94% of respondents were classified as having good knowledge, while 6% were classified as having poor knowledge (see Supplementary Data 2).

### Attitude and perception of PLHIV on antiretroviral therapy

Table 3 summarizes respondents’ attitudes and perceptions regarding antiretroviral therapy. A large proportion (87.3%, 448/513) considered ART to be the most effective treatment for HIV, and 76.6% expressed confidence in its effectiveness, although a minority (23.4%, 120/513) remained uncertain. Moreover, 15.6% believed that taking ART does more harm than good.

**Table 3:** Attitude and perception of PLHIV on antiretroviral therapy

Variables	Frequency (N=513)	Percentage (%)
Do you believe that there are other, more effective methods to treat HIV than using HIV drugs? *Perception*		
Yes	65	12.7
No	448	87.3
Are you convinced of the effectiveness of HIV drugs? *Attitude*		
Yes	393	76.6
No	120	23.4
Do you think that taking HIV drugs does more harm than good? *Attitude*		
Yes	80	15.6
No	433	84.4
Are you convinced that you should continue your HIV drugs? *Attitude*		
Yes	450	87.7
No	63	12.3
Have you ever felt ashamed to take your HIV drugs? - *Attitude*		
Yes	90	17.5
No	423	82.5
Do you think taking your HIV drugs is burdensome? *Perception*		
Yes	116	22.6
No	397	77.4

Most participants (87.7%, 450/513) regarded ART as both beneficial and necessary, yet 12.3% (63/513) reported that they did not believe they should continue using it. Emotional and psychological dimensions were also explored: 17.5% (90/513) of respondents reported feelings of shame associated with taking ART, and 22.6% (116/513) viewed the treatment as burdensome.

Using the scoring approach outlined in the methodology, the mean attitude score was 2.89, and the mean perception score was 0.18. Based on these thresholds, 85.6% of participants were classified as having a positive attitude toward ART, while 14.4% exhibited a negative attitude (see Supplementary Data 2). Similarly, 33.3% of respondents demonstrated a good perception of ART, whereas 66.7% were categorized as having a poor perception (see Supplementary Data 2).

### The practices of PLHIV towards antiretroviral therapy

Table 4 presents the practices of people living with HIV concerning their use of antiretroviral therapy. Nearly half of the respondents (45.4%, 233/513) reported storing their medications in a concealed location within their residence, while 21.4% (110/513) stored their drugs in a manner that helps them remember their daily dosing schedule. Regardless of storage location, most respondents (67.4%, 346/513) retained their medications in the original carton and plastic packaging.

**Table 4:** The practices of PLHIV towards antiretroviral therapy

Variables	Frequency (N=513)	Percentage (%)
Where do you store your HIV drugs at home?		
Hidden and out of sight of everyone	233	45.4
Convenient storage but not necessarily as recommended by my caregiver	40	7.8
Storage that can help to remember the daily schedule	110	21.4
Storage out of the reach and sight of children	48	9.4
Suitable storage as recommended by the manufacturer	82	16.0
How do you store your HIV drugs at home?		
Without its original carton packaging	93	18.1
Without its original plastic packaging	32	6.2
In other plastic packaging	42	8.2
In its original carton and plastic packaging	346	67.4
Have you at any time missed a dose of your HIV drugs?		
Yes	231	45.0
No	282	55.0
If yes, how often do you miss doses of your HIV drugs?		
Rarely	221	43.1
Frequently	10	1.9
Not applicable	282	55.0
During the last 7 days, how many times have you missed taking your HIV DRUGS?		
Once	80	15.6
Two times	15	2.9
More than two times	13	2.5
I did not miss it	405	78.9
How do you remember to take your HIV drugs?		
No particular method (habits)	320	62.4
Help from a relative	24	4.7
Reminder device	169	32.9
Have you ever increased or decreased the dose of your HIV drugs?		
Yes	47	9.2
No	466	90.8
Have you ever thrown away your HIV DRUGS?		
Yes	7	1.4
No	506	98.6
Have you ever lost or misplaced your HIV DRUGS?		
Yes	19	3.7
No	494	96.3
If your answer is YES to the above, what action did you take?		
I reported to my healthcare for a refill	13	68.4
I collected drugs from a friend	3	15.8
I stayed back till my next appointment date	3	15.8
I went to another healthcare facility to register as a new patient	0	0.0

Almost half (45.0%, 213/513) indicated that they had missed at least one dose of ART at some point in the past. However, 43.1% (221/513) stated that missing a dose was a rare occurrence, and the majority (78.9%, 405/513) reported not missing any doses in the seven days preceding the survey.

Regarding adherence support strategies, most respondents (62.4%, 320/513) reported not relying on any specific reminder method, noting that taking ART had become a habitual behavior. Nonetheless, a notable proportion (32.9%, 169/513) used reminder devices such as alarms to support adherence.

Most respondents (90.8%, 466/513) reported that they had never altered their dosage regimen, and 98.6% (506/513) had not discarded any of their medications. Similarly, most participants (96.3%, 494/513) had not reported losing or misplacing their ART. Among the 19 respondents who reported losing or misplacing their drugs, 13 (68.4%) reported to their healthcare provider for a refill, 3 (15.8%) collected drugs from a friend, 3 (15.8%) waited until their next appointment date, and none registered at another healthcare facility.

### Correlation between knowledge, attitude, and perception of PLHIV on antiretroviral therapy

Pearson correlation analysis (Table 5) revealed a statistically significant but weak negative correlation between knowledge and perception of ART (r(513) = −0.129, *p* = 0.003), as well as a weak positive correlation between attitude and perception (r(513) = 0.137, *p* = 0.002). No significant correlation was found between knowledge and attitude (r(513) = 0.018, *p* = 0.683). These results suggest that while knowledge and attitude may operate independently, perception is modestly associated with both.

**Table 5:** Pearson correlation between knowledge, attitude, and perception of PLHIV on antiretroviral therapy

Variable	Knowledge	Attitude	Perception
Knowledge of PLHIV			
Correlation Coefficient (r)	1	0.018	−0.129*
*P*-value		0.683	0.003
Attitude towards ART			
Correlation Coefficient (r)	0.018	1	0.137*
*P*-value	0.683		0.002
Perception towards ART			
Correlation Coefficient (r)	−0.129*	0.137*	1
*P*-value	0.003	0.002	

*Correlation is significant at *p* ≤ 0.05

### Multiple linear regression analysis of attitude and perception as predictors of knowledge level toward ART among PLHIV

Table 6 presents the results of the multiple linear regression analysis examining the effects of attitude and perception on the knowledge level (KL) of people living with HIV regarding antiretroviral therapy.

The analysis revealed that attitude did not have a statistically significant effect on knowledge level (β = 0.246, *p* = 0.412). However, perception showed a statistically significant negative association with knowledge level (β = − 0.609, *p* = 0.003), indicating that less favorable perceptions were associated with lower levels of knowledge about ART.

The model explained a small portion of the variance in knowledge level, with an R² = 0.018 and adjusted R² = 0.014, suggesting that attitude and perception together accounted for approximately 1.8% of the variance in knowledge level among respondents. Despite the modest explanatory power, the overall model was statistically significant (F(1, 512) = 4.642, *p* = 0.010).

**Table 6:** Multiple linear regression analysis of attitude and perception as predictors of knowledge level toward ART among PLHIV

Independent Variable	Unstandardized Coefficient (B)	Standard Error	t-value	*p*-value
Constant	5.569	0.157	35.372	0.001
Attitude	0.246	0.297	0.821	0.412
Perception	− 0.609	0.200	–3.019	0.003*

Model Summary

*R* = 0.134

R² = 0.018

Adjusted R² = 0.014

FF-statistics = 4.642

*p*-value (model) = 0.010

*Significant at *p* < 0.05

## Discussion

The potential positive impact of the scale-up of antiretroviral therapy is under threat from an increase in the prevalence of HIV drug resistance (HIVDR) [23]. In recent years, the factors driving HIVDR have been of great concern for physicians and stakeholders in the management of HIV. Suboptimal adherence to ART has long been identified as a major contributor to the development of HIVDR among people living with HIV [24, 25]. This emphasizes why knowledge, attitude, perception, and practice of PLHIV on antiretroviral therapy are crucial to identifying some of the factors responsible for poor adherence to ART and designing useful health education programs that could help to mitigate the emerging HIVDR.

This study reveals that more women (74.9%) participated in the survey compared to men (25.1%), aligning with findings from previous studies that also reported higher female participation in HIV-related research [26, 27]. This also emphasizes that more females are on antiretroviral drugs [28]. More so, in a country like Nigeria, where polygamy is widely practiced [29], there is a higher tendency for the disease to spread faster among women than men. The higher percentage of women could also mean that women are more willing to seek help and treatment than men.

This study found that the majority of PLHIV (94%) demonstrated good knowledge of their antiretroviral therapy—a finding that aligns with a cross-sectional study by Olowookere et al. (2012), where 75.2% of respondents similarly exhibited good knowledge of ART [17]. A qualitative study conducted among PLHIV in Uganda also noted that knowledge of ART drug combinations, the appropriate time to start taking ART, the benefits of taking ART regularly, and the possible results of not adhering to one’s medication was high among all participants [30].

In this study, nearly 65% of respondents could identify the names of their drug, with the majority identifying it as Tenofovir/Dolutegravir/Lamivudine. However, this contradicts the findings of Dzansi et al. (2020), which reported that most PLHIV could not mention the name of their medications, even though they had no difficulty identifying the drugs and understanding how they were to be taken [31]. This discrepancy may be attributed to the fact that most respondents in our study had a formal education (96.7%), which likely contributed to their ability to correctly identify their antiretroviral medications.

In addition, our results showed that approximately one-third (33.9%) of respondents were unable to recall the names of their medications. This has significant implications for HIV care. This may indicate gaps in patient health literacy and engagement, which are critical for optimal adherence and treatment outcomes. Studies have shown that patients with limited knowledge of their medications are more likely to experience poor adherence and medication errors, particularly when managing complex regimens or transitioning between care providers [32, 33]. Moreover, in many low-resource settings, ART is often dispensed in unlabeled packaging, and patients may identify drugs by physical characteristics rather than names, reflecting broader systemic issues in patient education and counseling [31]. Addressing these gaps through improved communication and patient empowerment strategies is essential to enhancing adherence and continuity of care.

Most respondents (81.9%) knew that ARTs are to be taken for life and understood that their purpose is to suppress the virus rather than to cure HIV. Such treatment literacy plays a critical role in supporting consistent adherence among PLHIV, which is vital for long-term viral suppression and preventing drug resistance [33, 34]. Our findings are consistent with a study that reported that 75.8% of PLHIV stated correctly that ART consists of drugs that suppress the activity of HIV [17].

High viral load and low CD4 + cell count are independently associated with increased mortality among PLHIV, and changes in these parameters during treatment are strongly linked to survival outcomes [35, 36]. Despite the clinical importance of monitoring these biomarkers, our findings reveal significant knowledge gaps. While 76.2% of respondents correctly identified that ART reduces viral load, nearly a quarter were unaware or misinformed. Similarly, only 45.6% understood ART’s role in improving CD4 + count, with over half either unsure or believing ART decreases it. These findings echo previous reports of limited understanding of viral load and CD4 + concepts among PLHIV in Nigeria [37, 38]. Strengthening patient education on the purpose and implications of these tests is essential for empowering treatment adherence and engagement.

Furthermore, our results show that the majority (81%) of the respondents had a satisfactory or good attitude toward antiretroviral therapy. However, a significant proportion (17.5%) of the respondents in this study indicated that they feel ashamed of taking their HIV drugs. This corroborates a previous study by Olowookere *et al. (2012)*, in which a slightly higher percentage (22.6%) of PLHIV reported that they felt it was shameful to be on ARVs [17]. These results indicate that stigma remains a barrier to HIV prevention and treatment. A study by Than et al. reveals that PLHIV reported different forms of stigmatization, such as shame, blame, and discrimination [39]. It is worth noting that more than 20% of the participants in this study declined to reveal their HIV status to people other than their healthcare givers. This attitude could have resulted from fear of stigmatization from every sector of Nigerian society, as reported in previous studies [40, 41]. Therefore, government policies should strongly discourage HIV stigmatization.

Achieving sustained viral suppression requires consistent daily adherence to antiretroviral therapy [42]. In this study, 22.6% of respondents reported that taking ART daily was burdensome, a finding consistent with previous research [43]. This perception may increase the risk of non-adherence and highlights the need for targeted interventions to address treatment fatigue and improve long-term adherence. Expanded access should be considered for drugs like Lenacapavir, a novel long-acting capsid inhibitor for HIV that can be added to the ARV regimen of heavily treatment-experienced PLHIV [44]. Lenacapavir requires a unique twice-a-year subcutaneous administration and has been proven to benefit PLHIV in achieving viral suppression and immune restoration [44].

In addition, nearly half (45.4%) of the participants in this study keep their ARV drugs hidden and out of sight. This practice also reflects a fear of stigmatization that was discussed earlier. Improper storage of drugs could have implications for their stability and active pharmaceutical components when stored in places with unsuitable temperature and moisture requirements. Forced degradation of drug substances can occur due to improper storage, and ARTs are no exception [45].

Our study found that 78.9% of respondents reported not missing any dose of their ART in the past seven days. While this suggests a generally favorable trend, it does not necessarily reflect the individual-level adherence standard recommended by the World Health Organization, which defines optimal adherence as taking at least 95% of prescribed doses over time [46]. Therefore, caution is needed when interpreting this result as meeting the WHO’s adherence threshold.

Moreover, the results of the multiple linear regression analysis indicate that respondents’ knowledge level did not have a statistically significant influence on their attitude toward antiretroviral therapy (ART). However, participants’ perceptions of ART showed a significant negative association with their knowledge levels. This suggests that unfavorable perceptions may hinder accurate understanding of ART. Similar findings have been documented in prior studies, which report that stigma, fear of side effects, and misinformation about ART often shape negative perceptions that persist even among individuals with some awareness of HIV treatment [47, 48]. Negative beliefs about ART—such as concerns about toxicity or doubts regarding its effectiveness—can undermine knowledge acquisition and interfere with treatment literacy [47]. In contexts where public education on ART is inconsistent or informal, such perceptions may stem more from community narratives than from formal healthcare interactions, thus limiting the impact of accurate knowledge. These findings highlight the importance of integrating perception-targeted interventions, including community-based health education and counseling, alongside conventional knowledge-based programs to improve both understanding and attitudes toward ART.

A major strength of this study lies in its multicenter design, which is the first of its kind to comprehensively assess knowledge, attitudes, perceptions, and practices toward antiretroviral therapy (ART) among people living with HIV across multiple secondary healthcare facilities in Lagos State, Nigeria. Unlike previous studies that were largely limited to single-site assessments or focused narrowly on specific components such as knowledge or adherence, this study provides a broader and more representative understanding of ART-related behaviors across diverse clinical settings and patient populations. This approach enhances the generalizability of the findings and allows for more robust insights into context-specific barriers and facilitators of ART uptake and adherence, thereby offering valuable evidence to inform targeted public health interventions and policy decisions within the region.

### Limitations of the study

Despite its strengths, this study relied solely on self-reported responses from participants, which are inherently subjective and may be prone to recall or social desirability bias. To address this, future research should consider incorporating objective measures to validate and complement self-reported data. Additionally, participants’ attitudes and perceptions were assessed using binary “yes” or “no” response options. While this approach provides a clear and straightforward indication of their views, it may not fully capture the complexity and depth of their opinions or experiences. A qualitative approach is therefore recommended for future studies to explore the nuanced dimensions of PLHIV’s attitudes and perceptions toward antiretroviral therapy.

Furthermore, although the study was designed to explore the interplay between knowledge, attitudes, perceptions, and practices (KAPP) regarding ART, only the knowledge, attitude, and perception components were quantitatively scored. The practice component was not scored and was consequently excluded from the regression and correlation analyses. As such, the analyses focused only on the relationships among knowledge, attitudes, and perceptions. This partial fulfillment of the study objective is acknowledged as a limitation and should be considered when interpreting the findings.

Additionally, the sample size was not stratified across the six study sites due to practical constraints such as variations in patient flow and logistical considerations. As such, proportional representation from each facility was not ensured. This may limit the ability to generalize findings to each site, and future studies may consider stratified or multilevel sampling to explore site-specific differences.

Lastly, the use of purposive sampling to select participating health facilities, while intentional to ensure inclusion of diverse local government areas with high ART patient loads, may introduce selection bias and limit the generalizability of the findings. Future studies should consider employing probabilistic sampling techniques to enhance representativeness and minimize potential bias in facility selection.

## Conclusion

This study highlights that while people living with HIV in Lagos State generally possess good knowledge about antiretroviral therapy, important gaps remain, particularly in their understanding of treatment monitoring indicators such as viral load and CD4 + cell counts. Furthermore, negative perceptions and persistent HIV-related stigma continue to undermine treatment literacy, medication adherence, and openness about HIV status, which are critical to long-term treatment success and the prevention of drug resistance.

Our findings emphasize the need for targeted public health interventions that address not only knowledge gaps but also the social and psychological barriers affecting ART uptake and adherence. Strengthening community education, combating stigma, and exploring adherence-supportive innovations such as long-acting ART formulations should be prioritized to improve health outcomes among PLHIV and support broader HIV control efforts in Nigeria.

## Supplementary Information

Supplementary Material 1.

Supplementary Material 2.

## Data Availability

All data generated or analyzed during this study are included in this published article [and its supplementary information files].

## Abbreviations

WHO: World Health Organization
HIV: Human Immunodeficiency Virus
PLHIV: People Living With HIV
AIDS: Acquired Immunodeficiency Syndrome
ART: Antiretroviral therapy
KAPP: Knowledge, attitudes, perceptions, and practices
HAART: Highly active antiretroviral therapy
HIVDR: HIV drug resistance

## References

[1] World Health Organization HIV and AIDS Fact Sheets. 2024 [Accessed on June 30, 2025]. https://www.who.int/news-room/fact-sheets/detail/hiv-aids

[2] Oreagba LA, Usman SO, Olayemi SO, Oshikoya KA, Opanuga O, Adeyemo TA, Lesi OA, Dodoo AN, Akanmu AS. Pharmacoepidemiology of antiretroviral drugs in a teaching hospital in Lagos, Nigeria. Ghana Med J. 2014;48(4):194–203. 25709134 10.4314/gmj.v48i4.5 PMC4335432

[3] Global Health Observatory Data Repository (GHO)|. By category | Number of people (all ages) living with HIV - Estimates by WHO region [https://apps.who.int/gho/data/node.main.620?lang=en].

[4] Adekunjo, Felix O. Contingent Valuation and Determinants of HIV Counselling and Testing Service in Lagos State of Nigeria 2018. Available from ProQuest Dissertations & Theses Global. (2877960842). http://libproxy.usouthal.edu/login?url=https://www.proquest.com/dissertations-theses/contingent-valuation-determinants-hiv-counselling/docview/2877960842/se-2.

[5] Joseph AA, Joseph OA, Olokoba BL, Olatunji OA. Chronicles of challenges confronting HIV prevention and treatment in Nigeria. Port Harcourt Med J. 2020;14(3):100–13.

[6] Baveewo S, Ssali F, Karamagi C, Kalyango JN, Hahn JA, Ekoru K et al. Validation of World Health Organisation HIV/AIDS Clinical Staging in Predicting Initiation of Antiretroviral Therapy and Clinical Predictors of Low CD4 Cell Count in Uganda. Semple MG, editor. PLoS ONE. 2011;6(5):e19089. 10.1371/journal.pone.0019089 PMC3093378 21589912

[7] McIntyre AF, Mitchell A, Stafford KA, Nwafor SU, Lo J, Sebastian V, et al. Key population size estimation to guide HIV epidemic responses in Nigeria: bayesian analysis of 3-source capture-recapture data. JMIR Public Health Surveill. 2022;8(10):e34555. 36287587 10.2196/34555 PMC9647455

[8] Kalichman SC, Cherry J, Cain D. Nurse-delivered antiretroviral treatment adherence intervention for people with low literacy skills and living with HIV/AIDS. J Assoc Nurses AIDS Care. 2005;16(5):3–15. 16433105 10.1016/j.jana.2005.07.001

[9] World Health Organization. Consolidated guidelines on the use of antiretroviral drugs for treating and preventing HIV infection: recommendations for a public health approach. Geneva: World Health Organization. 2013. 269 p. Available from: https://iris.who.int/handle/10665/85321. Cited 2025 Jun 30. 24716260

[10] Umeokonkwo CD, Onoka CA, Agu PA, Ossai EN, Balogun MS, Ogbonnaya LU. Retention in care and adherence to HIV and AIDS treatment in Anambra state Nigeria. BMC Infect Dis. 2019;19(1):654. 31331280 10.1186/s12879-019-4293-8 PMC6647106

[11] Bright Asuquo E. An exploration of approaches employed to improve ART adherence among people living with HIV in Nigeria. 2023. (Master thesis, KIT (Royal Tropical Institute) Vrije Universiteit Amsterdam (VU)). Available from: http://bibalex.org/baifa/Attachment/Documents/nBpnG0rASO_20231127121921527.pdf.

[12] Ezenduka J, Nwaokennaya P, Obasa GB, Ogbeke G, Onuorah O, Abubakar L, et al. Perception of people living with HIV and healthcare workers on differentiated service delivery programs in nigeria: A qualitative Study. Aworh MK. Editor PLoS One. 2025;20(5):e0309254. 10.1371/journal.pone.0309254 PMC12111639 40424221

[13] Campbell C, Nair Y, Maimane S, Nicholson J. `Dying twice’: a multi-level model of the roots of AIDS stigma in two South African communities. J Health Psychol. 2007;12(3):403–16. 17439992 10.1177/1359105307076229

[14] Dahlui M, Azahar N, Bulgiba A, Zaki R, Oche OM, Adekunjo FO et al. HIV/AIDS Related Stigma and Discrimination against PLWHA in Nigerian Population. Kumar A, editor. PLoS ONE. 2015;10(12):e0143749. 10.1371/journal.pone.0143749 PMC4675522 26658767

[15] Apena WO. A knowledge-based HIV/AIDS framework for Lagos State. 2012 (Doctoral dissertation, Coventry University). Available from: https://www.academia.edu/download/92890532/402345321.pdf.

[16] Oguh CE, Obiwulu ENO, Sheshi IM, Ameh SE, Okpaka CO, Oluwadepo TJ et al. The epidemiology pattern of human immunodeficiency Virus/Acquire immune deficiency Syndrome, Diagnostic, transmission and prevention in Nigeria-Past and present. 2021;29-50. http://scholar.google.com/scholar_lookup?&title=The%20epidemiology%20pattern%20of%20human%20immunodeficiency%20virus%2Facquire%20immune%20deficiency%20syndrome%2C%20diagnostic%2C%20transmission%20and%20prevention%20in%20Nigeria-past%20and%20present&journal=Asian%20J%20Res%20Infect%20Dis&volume=6&pages=29-50&publication_year=2021&author=Oguh%2CC&author=Obiwulu%2CE&author=Sheshi%2CI&author=Ameh%2CS&author=Okpaka%2CC&author=Oluwadepo%2CT&author=Ejiofor%2CU.

[17] Olowookere SA, Fatiregun AA, Adewole IF. Knowledge and attitudes regarding HIV/AIDS and antiretroviral therapy among patients at a Nigerian treatment clinic. J Infect Dev Ctries. 2012;6(11):809–16. 23277507 10.3855/jidc.2086

[18] Eze RA, Sulaiman N, Mat Daud Z, ’Azuan BA. Association Between Belief in Medicine and Adherence to Antiretroviral Therapy Among Human Immunodeficiency Virus Adults in Zaira, Kaduna State, Nigeria. Cureus. 2023; Available from: https://www.cureus.com/articles/144241-association-between-belief-in-medicine-and-adherence-to-antiretroviral-therapy-among-human-immunodeficiency-virus-adults-in-zaira-kaduna-state-nigeria. Cited 2025 Jun 27. 10.7759/cureus.36489 PMC10118400 37090307

[19] Uakarn C. Sample size estimation using Yamane and Cochran and Krejcie and Morgan and green formulas and Cohen statistical power analysis by G*power and comparisons. Apheit Int J. 2021;10(2):76–88.

[20] Raberahona M, Lidamahasolo Z, Andriamamonjisoa J, Andriananja V, Andrianasolo RL, Rakotoarivelo RA, et al. Knowledge, attitudes, perception and practices regarding antiretroviral therapy among HIV-infected adults in Antananarivo, Madagascar: a cross-sectional survey. BMC Health Serv Res. 2019;19(1):341. 31138303 10.1186/s12913-019-4173-3 PMC6537363

[21] Olum R, Chekwech G, Wekha G, Nassozi DR, Bongomin F. Coronavirus disease-2019: knowledge, attitude, and practices of health care workers at Makerere university teaching Hospitals, Uganda. Front Public Health. 2020;8:181. 32426320 10.3389/fpubh.2020.00181 PMC7204940

[22] Ejeh FE, Saidu AS, Owoicho S, Maurice NA, Jauro S, Madukaji L, et al. Knowledge, attitude, and practice among healthcare workers towards COVID-19 outbreak in Nigeria. Heliyon. 2020;6(11):e05557. 33230488 10.1016/j.heliyon.2020.e05557 PMC7673666

[23] Beyrer C, Pozniak A. HIV drug resistance — an emerging threat to epidemic control. N Engl J Med. 2017;377(17):1605–7. 29069566 10.1056/NEJMp1710608

[24] HIV Drug Resistance Report. 1st ed. Geneva: World Health Organization. 2021. Available from: https://www.who.int/publications/i/item/9789240038608. Accessed 17 November 2025.

[25] Bangsberg DR, Acosta EP, Gupta R, Guzman D, Riley ED, Harrigan PR, et al. Adherence–resistance relationships for protease and non-nucleoside reverse transcriptase inhibitors explained by virological fitness. AIDS. 2006;20(2):223–31. 16511415 10.1097/01.aids.0000199825.34241.49

[26] Lawal TV, Oyedele OK, Andrew NP. On characterizing gender and locational composition of adult PLHIV in Nigeria: Implications for HIV programming. Robinson J, editor. PLOS Glob Public Health. 2024;4(8):e0002863. 10.1371/journal.pgph.0002863 PMC11346663 39186499

[27] Charles-Eromosele O, Kanma-Okafor TJ, O Sekoni O, O Olopade A, Olopade BB, Ekanem OE. Gender disparities in the socio-economic burden of HIV/AIDS among patients receiving care in an HIV clinic in Lagos, Nigeria. Afr H Sci. 2022;22(4):477–87. 10.4314/ahs.v22i4.54 PMC10117501 37092064

[28] Muula AS, Ngulube TJ, Siziya S, Makupe CM, Umar E, Prozesky HW, et al. Gender distribution of adult patients on highly active antiretroviral therapy (HAART) in Southern Africa: a systematic review. BMC Public Health. 2007;7(1):63. 17459154 10.1186/1471-2458-7-63 PMC1868718

[29] Kramer S, Hackett C, Schiller A, Nolan H. Religion and living arrangements around the world. Religion and living arrangements around the world. Pew Res Centre. 2019. Available from: https://www.pewresearch.org/religion/2019/12/12/religion-and-living-arrangements-around-the-world/. Accessed 17 November 2025.

[30] Weiser SD, Tuller DM, Frongillo EA, Senkungu J, Mukiibi N, Bangsberg DR. Food Insecurity as a Barrier to Sustained Antiretroviral Therapy Adherence in Uganda. Grietens KP, editor. PLoS ONE. 2010;5(4):e10340. 10.1371/journal.pone.0010340 PMC2860981 20442769

[31] Dzansi G, Tornu E, Chipps J. Promoters and inhibitors of treatment adherence among HIV/AIDS patients receiving antiretroviral therapy in Ghana: narratives from an underserved population. PLoS One. 2020;15(3):e0230159. 32142549 10.1371/journal.pone.0230159 PMC7059913

[32] Ahmed AM, Sisay AL, Gebre MN. Incidence and predictors of loss to follow-up among adult HIV patients attending antiretroviral therapy at public health facilities in Agaro town, Southwest Ethiopia, 2023. BMC Infect Dis. 2025;25(1):297. 40025429 10.1186/s12879-025-10646-7 PMC11872337

[33] Kalichman SC, Pope H, White D, Cherry C, Amaral CM, Swetzes C, et al. Association between health literacy and HIV treatment adherence: further evidence from objectively measured medication adherence. J Int Assoc Physicians AIDS Care (Chic Ill). 2008;7(6):317–23. 10.1177/1545109708328130 PMC3015090 19056866

[34] Langebeek N, Gisolf EH, Reiss P, Vervoort SC, Hafsteinsdóttir TB, Richter C, Sprangers MA, Nieuwkerk PT. Predictors and correlates of adherence to combination antiretroviral therapy (ART) for chronic HIV infection: a meta-analysis. BMC medicine. 2014;12(1):142. 10.1186/s12916-014-0142-1 PMC4148019 25145556

[35] Jordan MR, Penazzato M, Cournil A, Vubil A, Jani I, Hunt G, Carmona S, Maphalala G, Mthethwa N, Watera C, Kaleebu P. Human immunodeficiency virus (HIV) drug resistance in African infants and young children newly diagnosed with HIV: a multicountry analysis. Clin Infect Dis. 2017;65(12):2018–25. 29020335 10.1093/cid/cix698

[36] Nsanzimana S, Remera E, Kanters S, Forrest JI, Ford N, Condo J, et al. Effect of baseline CD4 cell count at linkage to HIV care and at initiation of antiretroviral therapy on mortality in HIV-positive adult patients in Rwanda: a nationwide cohort study. Lancet HIV. 2015;2(9):e376-84. 26423551 10.1016/S2352-3018(15)00112-5

[37] Chidera IV, D. IE MIO, Jeremiah IC, Ngozi OS, Amaechi EC, et al. Determinants of adherence to antiretroviral therapy among HIV patients in a tertiary healthcare facility in southeastern Nigeria. Int J Trop Dis Health. 2025;46(4):28–44.

[38] Adeniran A, Shogbamimu Y, Ojo OY, Chieme FC, Olowofeso HO, Sidebe I, et al. How do people living with HIV (PLHIV) and AIDS feel about the quality of care they received amid the COVID-19 pandemic in Lagos, Nigeria? J Int Assoc Provid AIDS Care. 2023;22:23259582231196708. 37635327 10.1177/23259582231196708 PMC10467289

[39] Than PQT, Tran BX, Nguyen CT, Truong NT, Thai TPT, Latkin CA, et al. Stigma against patients with HIV/AIDS in the rapid expansion of antiretroviral treatment in large drug injection-driven HIV epidemics of Vietnam. Harm Reduct J. 2019;16(1):6. 30654814 10.1186/s12954-019-0277-7 PMC6337792

[40] Adimora DE, Aye EN, Akaneme IN, Nwokenna EN, Akubuilo FE. Stigmatization and discrimination as predictors of self-esteem of people living with HIV and AIDS in Nigeria. Afr Health Sci. 1970;19(4):3160–71. 10.4314/ahs.v19i4.39 PMC7040355 32127893

[41] Olley BO, Ogunde MJ, Oso PO, Ishola A. HIV-related stigma and self-disclosure: the mediating and moderating role of anticipated discrimination among people living with HIV/AIDS in Akure Nigeria. AIDS Care. 2016;28(6):726–30. 26882476 10.1080/09540121.2016.1140894

[42] Bangsberg DR, Perry S, Charlebois ED, Clark RA, Roberston M, Zolopa AR, et al. Non-adherence to highly active antiretroviral therapy predicts progression to AIDS. AIDS. 2001;15(9):1181–3. 11416722 10.1097/00002030-200106150-00015

[43] Schreiner N, Perazzo J, Currie J, Daly B, Webel A. A descriptive, cross-sectional study examining treatment burden in people living with HIV. Appl Nurs Res. 2019;46:31–6. 30853073 10.1016/j.apnr.2019.02.009 PMC6746227

[44] Tailor MW, Chahine EB, Koren D, Sherman EM. Lenacapavir: a novel Long-Acting capsid inhibitor for HIV. Ann Pharmacother. 2024;58(2):185–95. 37138515 10.1177/10600280231171375

[45] Lamichhane S, Das B, Adhikari RP, Jeyaprakash MR. Overview of forced degradation analysis for FDA approved antiretroviral agents: a review. J Young Pharm. 2022;14(3):273–82.

[46] Frescura L, Godfrey-Faussett P, Feizzadeh A, El-Sadr W, Syarif O, Ghys P et al. Z Ambrose editor 2022 Achieving the 95 95 95 targets for all: A pathway to ending AIDS. PLoS ONE 17 8 e0272405. 35925943 10.1371/journal.pone.0272405 PMC9352102

[47] Kalichman SC, Eaton L, Cherry C. There is no proof that HIV causes AIDS: AIDS denialism beliefs among people living with HIV/AIDS. J Behav Med. 2010;33(6):432–40. 20571892 10.1007/s10865-010-9275-7 PMC3015095

[48] Peltzer K, Friend-du Preez N, Ramlagan S, Anderson J. Antiretroviral treatment adherence among HIV patients in KwaZulu-Natal, South Africa. BMC Public Health. 2010;10(1):111. 20205721 10.1186/1471-2458-10-111 PMC2837855

