# Supplementary material for: Knowledge, attitudes, perceptions, and practices toward antiretroviral therapy among people living with HIV in secondary healthcare facilities in Lagos State, Nigeria: a multicenter cross-sectional study
Source: BMC Public Health. 2025 Dec 30;25:4367. doi: 10.1186/s12889-025-25629-1 (PMC12754896; doi:10.1186/s12889-025-25629-1)
Supplement: Supplementary file 1 — Supplementary Material 1. [file 12889_2025_25629_MOESM1_ESM.docx]

**QUESTIONNAIRE**

This is a study designed to assess the knowledge, awareness, perception and practices of PLHIV on antiretroviral drugs in Lagos, Nigeria. It is sponsored by the Association of Hospital and Administrative Pharmacists of Nigeria, Lagos and has been approved by the Health, Research and Ethics Committee of Lagos State University Teaching Hospital (LASUTH) (ref No: LREC/06/10/2191)

**YOUR PARTICIPATION IN THIS STUDY IS VOLUNTARY**

Do you consent to take part in this study?

☐ YES ☐ NO

| **SOCIO-DEMOGRAPHIC DATA** | |
| --- | --- |
| 1. **Gender** | ☐ Male ☐ Female |
| 1. **Marital status** | ☐ Single ☐ Married ☐ Divorced ☐ Widowed  ☐ Separated |
| 1. **Age at last birthday (years)** |  |
| 1. **Residence in Lagos (Urban, semi-urban, rural)** |  |
| 1. **Sexual orientation** | ☐ Heterosexual ☐ Homosexual ☐ Bisexual |
| 1. **Profession** | ☐ Student ☐ Unemployed  ☐ Employed ☐ Retired |
| 1. **Level of education** | ☐ No formal education ☐ Primary  ☐ Secondary ☐ Graduate  ☐ Postgraduate |
| 1. **How long have you been using HIV drug?** | ___/____ (month/year) |
| 1. **Are you living alone (if yes, go to question 11)** | ☐ Yes ☐ No |
| 1. **How many people are you living with?** |  |
| 1. **Have you disclosed your HIV status with anyone other than your healthcare providers?**   **(If no, go to question 13)** | ☐ Yes ☐ No |
| 1. **If yes, who did you disclose it to?** | ☐ Spouse ☐ Mother ☐ Father ☐ Brother  ☐ Children ☐ Friends ☐ Sexual partner  ☐ Sister ☐ Other (please specify) ___________ |
| **KNOWLEDGE OF ANTIRETROVIRAL THERAPY** | |
| 1. **What is the name of your HIV drugs?** | ☐ TENOFOVIR/DOLUTEGRAVIR/LAMIVUDINE  ☐ ABACAVIR/LAMIVUDINE/DOLUTEGRAVIR  ☐ TENOFOVIR/LAMIVUDINE/ATAZANAVIR  ☐ ABACAVIR/LAMIVUDINE/ATAZANAVIR  ☐ ZIDOVUDINE/LAMIVUDINE/ATAZANAVIR  ☐ OTHERS (PLS, SPECIFY) ___________________  ☐ I DON’T KNOW IT |
| 1. **How many times should you take your HIV DRUGS in a day?** | ☐ once a day  ☐ twice daily  ☐ others (pls, mention) _______________  ☐ I don’t know |
| 1. **How long should you take your HIV DRUGS?** | ☐ AS LONG AS I LIVE  ☐ FOR SOME TIME ONLY  ☐ I don’t know |
| 1. **What is the purpose of HIV DRUGS?** | ☐ To suppress the activity of HIV but do not cure  ☐ It cures HIV/AIDS ☐ I don’t know |
| 1. **What is the effect of HIV DRUGS on HIV viral load?** | ☐ It increases viral load  ☐ it decreases viral load  ☐ No effect on viral load  ☐ I don’t know |
| 1. **What is the effect of HIV DRUGS on CD4 count?** | ☐ It increases CD4 count  ☐ it decreases CD4 count  ☐ No effect on CD4 load  ☐ I don’t know |
| 1. **HIV DRUGS are relatively safe to use** | ☐ Yes ☐ No |
| **ATTITUDE AND PERCEPTION OF ANTIRETROVIRAL THERAPY** | |
| 1. **Do you believe that there are other more effective methods to treat HIV than using HIV DRUGS? *perception*** | ☐ Yes ☐ No |
| 1. **Are you convinced of the effectiveness of HIV DRUGS?** | ☐ Yes ☐ No |
| 1. **Do you think that taking HIV DRUGS does more harm than good** | ☐ Yes ☐ No |
| 1. **Are you convinced that you should continue your HIV DRUGS?** | ☐ Yes ☐ No |
| 1. **Have you ever felt ashamed to take your HIV drugs?** | ☐ Yes ☐ No |
| 1. **Do you think taking your HIV drugs is burdensome? *perception*** | ☐ Yes ☐ No |
| **PRACTICE REGARDING ANTIRETROVIRAL THERAPY** | |
| 1. **Where do you store your HIV DRUGS at home?** | ☐ Hidden and out of sight of everyone  ☐ Convenient storage, but not necessarily as recommended by my caregiver  ☐ Storage that can help to remember daily schedule  ☐ Storage out of the reach and sight of children  ☐ Suitable storage as recommended by the manufacturer |
| 1. **How do you store your HIV DRUGS at home?** | ☐ Without its original carton packaging  ☐ Without its original plastic packaging  ☐ In other plastic packaging  ☐ In its original carton and plastic packaging |
| 1. **Have you at any time missed a dose of your HIV DRUGS?** | ☐ Yes ☐ No |
| **If yes, how often?** | ☐ Rarely ☐ Frequently |
| 1. **During the last 7 days, how many times have you missed taking your HIV DRUGS?** | ☐ once ☐ two times  ☐ more than two times ☐ I did not miss it |
| 1. **How do you remember to take your HIV DRUGS?** | ☐ No particular method (habits)  ☐ Help from a relative  ☐ Reminder device  ☐ Other (pls, specify): ________________ |
| 1. **Have you ever increased or decreased the dose of your HIV DRUGS?** | ☐ Yes ☐ No |
| 1. **Have you ever thrown away your HIV DRUGS?** | ☐ Yes ☐ No |
| 1. **Have you ever lost or misplaced your HIV DRUGS?** | ☐ Yes ☐ No |
| 1. **If your answer is YES to Q 33, what action did you take?** | ☐ I reported to my healthcare for a refill  ☐ I collected drugs from a friend  ☐ I stayed back till my next appointment date  ☐ I went to another healthcare facility to register as new patient  ☐ Not applicable |

Thank you for participating in this study. AHAPN Research Team
