# Supplementary material for: Knowledge, attitudes, perceptions, and practices toward antiretroviral therapy among people living with HIV in secondary healthcare facilities in Lagos State, Nigeria: a multicenter cross-sectional study
Source: BMC Public Health. 2025 Dec 30;25:4367. doi: 10.1186/s12889-025-25629-1 (PMC12754896; doi:10.1186/s12889-025-25629-1)
Supplement: Supplementary file 2 — Supplementary Material 2. [file 12889_2025_25629_MOESM2_ESM.docx]

**Clarification on Attitude and Perception Items**

In the questionnaire section titled *“Attitude and Perception of Antiretroviral Therapy,”* a combined set of six items was used to assess both constructs. While the items were presented under a single section for practicality, they were analyzed separately based on theoretical distinctions:

- Attitude refers to participants’ evaluative beliefs or convictions regarding antiretroviral therapy (ART).
- Perception refers to subjective interpretations or emotional responses toward ART.

The items and their assigned domains are listed below:

| **ATTITUDE AND PERCEPTION OF ANTIRETROVIRAL THERAPY** | |
| --- | --- |
| 1. **Do you believe that there are other more effective methods to treat HIV than using HIV DRUGS? *(perception)*** | ☐ Yes ☐ No |
| 1. **Are you convinced of the effectiveness of HIV DRUGS? *(attitude)*** | ☐ Yes ☐ No |
| 1. **Do you think that taking HIV DRUGS does more harm than good *(attitude)*** | ☐ Yes ☐ No |
| 1. **Are you convinced that you should continue your HIV DRUGS? *(attitude)*** | ☐ Yes ☐ No |
| 1. **Have you ever felt ashamed to take your HIV drugs? *(attitude)*** | ☐ Yes ☐ No |
| 1. **Do you think taking your HIV drugs is burdensome? *(perception)*** | ☐ Yes ☐ No |

**Table 1: Descriptive statistics for mean scores of the respondents’ Knowledge, Attitude, and Perception of their Antiretroviral therapy.**

| **Outcome variable** | **Maximum obtainable score** | **Mean ± SD** | **Good n (%)** | **Poor n (%)** |
| --- | --- | --- | --- | --- |
| Knowledge | 7 | 5.58 **±** 1.25 | 481 (94) | 32 (6) |
| Attitude | 4 | 2.89 **±** 0.18 | 416 (81.1) | 97 (18.9) |
| Perception | 2 | 0.18 ± 0.28 | 162 (31.6) | 351 (68.4) |


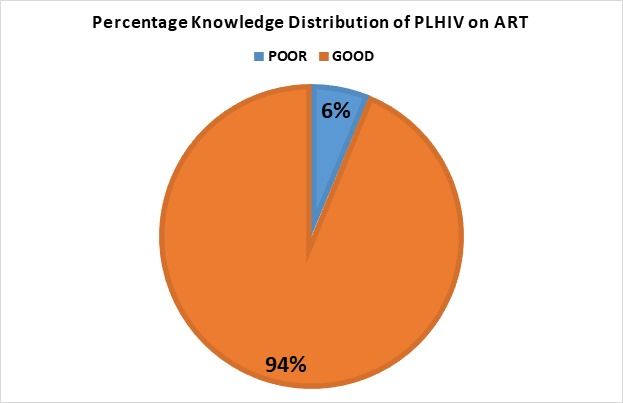


**Figure 1: Percentage Knowledge distribution of PLHIV on ART**


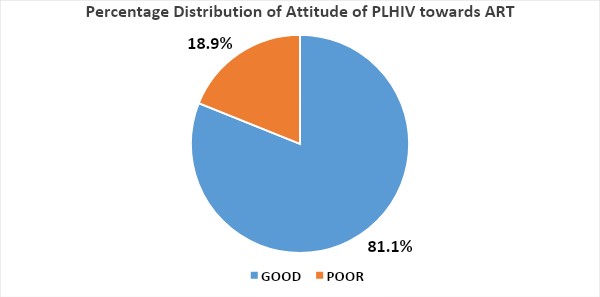


**Figure 3: Percentage distribution of attitude of PLHIV toward ART**


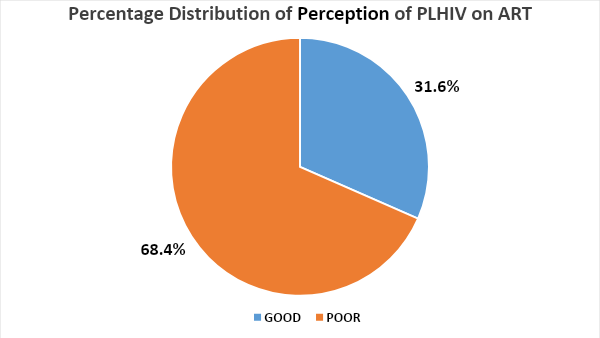


**Figure 4: Percentage distribution of perception of PLHIV on ART**
